# Molecular and socioeconomic characteristics of inflammatory breast cancer in the Carolina Breast Cancer Study

**DOI:** 10.1007/s10549-025-07884-3

**Published:** 2026-01-22

**Authors:** Qichen Wang, Sarah C. Van Alsten, Xiaojia Ji, Esraa Salim, Nicole Salazar, John E. Scott, Xiaohe Yang, Rob U. Onyenwoke, Melissa A. Troester, Kevin P. Williams

**Affiliations:** 1 Department of Epidemiology, Gillings School of Public Health, University of North Carolina at Chapel Hill, Chapel Hill, NC 27599 USA; 2 Lineberger Comprehensive Cancer Center, University of North Carolina at Chapel Hill, Chapel Hill, NC 27599 USA; 3 Biomanufacturing Research Institute and Technology Enterprise, North Carolina Central University, Durham, NC 27707 USA; 4 Julius L. Chambers Biomedical/Biotechnology Research Institute, North Carolina Central University, Durham, NC 27707 USA; 5 Department of Biological and Biomedical Sciences, North Carolina Central University, Durham, NC 27707 USA; 6 Department of Pharmaceutical Sciences, North Carolina Central University, Durham, NC 27707 USA; 7 Department of Pathology and Laboratory Medicine, University of North Carolina at Chapel Hill, Chapel Hill, NC 27599 USA

**Keywords:** Inflammatory Breast Cancer, PAM50, Socioeconomic Status, Carolina Breast Cancer Study

## Abstract

**Purpose:**

Inflammatory breast cancer (IBC) has been hypothesized to represent a distinct molecular subtype. However, few IBC-specific gene expression patterns have been identified, and previous genomic studies of IBC have been small with limited information on social determinants.

**Methods:**

We identified 153 IBC cases in the Carolina Breast Cancer Study (total *N* = 4,739). RNA expression was measured on the NanoString platform (*N* = 74 IBC, 2,696 non-IBC) and used to determine molecular subtypes, including PAM50, immune, homologous recombination deficiency (HRD), and P53 status. We estimated odds ratios (ORs) and 95% confidence intervals (CIs) of associations between IBC and patient demographic, molecular, and social characteristics using logistic regression, and compared differences in gene expression using ANOVA.

**Results:**

Women with IBC were associated with Black and under 50 compared to non-IBC. IBC was associated with rural address (OR = 1.53) and poverty (OR = 1.61). Molecularly, IBC was associated with HER2-enriched (OR = 6.14), Luminal B (OR = 2.90), P53 Mutant-like (OR = 1.79), and high HRD (OR = 1.90). Neither adiposity nor immune subtype was significantly associated with IBC. Only six of 219 genes measured were significantly differentially expressed between IBC and non-IBC, including HER2-related (ERBB2, FGFR4, GRB7) and P53-related genes (BTG2, LOC400043, MAP2K4).

**Conclusion:**

Although not associated with immune subtypes, IBC showed differences in HER2 and P53 pathways. The association of IBC with rurality and poverty underscores the importance of health care access for timely diagnosis and treatment of IBC.

**Supplementary Information:**

The online version contains supplementary material available at 10.1007/s10549-025-07884-3.

## Introduction

Inflammatory breast cancer (IBC) is a rare (∼1–5% of cases) yet highly aggressive and deadly breast cancer sub type [1, 2], that is challenging to diagnose [3, 4]. In IBC, the affected breast presents with rapid-onset redness, swelling, and warmth, symptoms which mimic an acute infection [5]. Hence, the label “inflammatory” may be misleading, as the hallmark erythema and edema result from carcinoma cells obstructing dermal lymphatic vessels rather than from any true infection or immune-driven inflammation [6]. Due to its unique clinical and molecular characteristics, it has been proposed that IBC is a separate entity from non-IBC breast cancer [7]. Pathologically, IBC typically shows minimal inflammatory cell infiltrate, and it produces only negligible levels of classic pro-inflammatory cytokines (e.g. IL-1, IL-12, IFN-γ) [8, 9]. This inconsistency in terminology highlights a knowledge gap in distinguishing IBC and non-IBC. Transcriptome-wide gene expression studies have aimed to address this gap by seeking a unique IBC molecular signature. However, the findings from these investigations remain inconsistent [10]. While some research has successfully identified distinct gene sets capable of differentiating IBC from non-IBC [11–14], other studies that aimed to validate these sets reported no significant distinctions between the two [15, 16]. Therefore, it remains unclear whether tumor gene expression can distinguish IBC from non-IBC.

Beyond molecular profiles, understanding the demographic and socioeconomic characteristics of IBC is important for contextualizing prognosis and outcome disparities. The aggressive tumor biology observed in IBC may occur in conjunction with population-level risk patterns. For example, higher rates of IBC in Black women have been reported [2, 17–21]. The interplay between socioeconomic status, geographic location, and IBC risk remains poorly understood, with conflicting reports on the association between IBC and factors such as education level, income, and urban versus rural residence [43, 44, 65–70]. In addition, few studies have comprehensively compared the social and molecular characteristics of IBC with non-IBC.

To address these gaps, this study used the Carolina Breast Cancer Study (CBCS, 1993–2013) which oversampled breast cancers from Black and younger women. RNA-based assays, demographic, and social factors were collected, providing a robust framework to examine both molecular and socioeconomic factors associated with IBC. We aimed to elucidate the factors associated with IBC, considering both tumor biology and access to care. Further, leveraging the large-scale, population-based design of the CBCS, this study provided a unique single resource spanning molecular, demographic, clinical, and socioeconomic data to allow a comprehensive comparison of IBC and non-IBC cases.

### Methods

#### Study population

The CBCS is a three-phase population-based study that recruited participants with ages between 20 and 74 from 44 counties in North Carolina (Phase I from 1993 to 1996, Phase II from 1996 to 2001, and Phase III from 2008 to 2013 [22–24]). Black and younger (< 50 years old) women were oversampled for CBCS using random recruitment [22]. In total, 4,806 invasive breast cancers were enrolled in the CBCS (Phase I-III, 2,283 Black and 2,523 non-Black). Among all, IBCs were identified according to medical records as follows: for CBCS Phase III, tumor stage was available from medical records, and IBC cases were identified if the tumor stage was inflammatory carcinoma. For CBCS Phase I and II, we relied on clinical descriptors that correspond to inflammatory breast cancer, notably as tumors of any size with direct extension to the chest wall or skin and involvement of skin or chest wall. These differences reflect greater appreciation for IBC as a clinical phenotype in 2008–2013 (Phase III) vs. 1993–2001 (Phase I-II). After excluding 67 cases with unknown IBC status, 4,739 cases remained for analysis, and identified 153 IBC (3%; 31 from Phase I, 55 from Phase II, and 67 from Phase III) and 4,586 non-IBC. All IBCs were stage (American Joint Committee on Cancer) III or IV, and 86% of non-IBCs were stage I or II.

### Demographic, clinical, and socioeconomic covariates

Demographic information was collected by a nurse during in-home interviews, and body mass index (BMI) and waist-to-hip ratio (WHR) were measured. We dichotomized BMI as less than 30 kg/m^2^ (YES) or not (NO), and grouped study participants into WHR < 0.85 (Yes) or not (No) to indicate obesity among women according to the World Health Organization [25]. Self-reported race was classified as Black and non-Black, with less than 5% of non-Black participants self-reporting as non-White (*n* = 21 American Indian or Eskimo, n = 43 Asian or Pacific Islander, and *n* = 42 Others). Human epidermal growth factor receptor 2 (HER2) status, estrogen receptor (ER) status, progesterone receptor (PR) status, and tumor grade were collected from medical records, pathology reports, and IHC staining was done at University of North Carolina at Chapel Hill (UNC-CH). We used a 10% positivity cutoff for hormone receptor (HR), consistent with prior reports suggesting 10% ER is the more etiologically relevant [26]. ER, PR, and HER2 were grouped into HR/HER2 status, where HR positive is defined as any of the ER or PR positive, and negative is defined otherwise. Tumor grade is available only partially for CBCS Phase I and III. The missing grade for Phase I, II, and III (*n* = 930) were imputed using the Multivariate Imputation by Chained Equations package [27] based on a previously published method, which used ER/PR/HER2 status, node status, race, age, tumor stage, size, p53 mutation status, survival, grade, and study phase as predictor variables [28].

Socioeconomic covariates were also collected during the in-home interviews, including education level (high school or less vs. above high school) and urban or rural residency (defined based on Rural–urban Continuum Codes at diagnosis), and were analyzed in this study. Additionally, we defined below 200% of the poverty level using family income and home size of participants according to the federal poverty guidelines from the United States Department of Health and Human Services [29] in the year of diagnosis.

### CBCS RNA processing and normalization

Of 4,806 invasive breast cancer cases in the CBCS, 1,188 were excluded from RNA analyses due to tissue depletion and 241 due to low RNA quality. RNA expression profiling with a customized NanoString panel was conducted for 2,783 of the remaining 3,377 cases. After excluding 13 participants with unknown IBC status, 2,770 cases (74 IBC and 2,696 non-IBC) were included in the molecular analyses.

RNA for CBCS samples was extracted from formalin-fixed, paraffin embedded bulk tumor tissue and profiled using the NanoString nCounter assay. Samples were randomized into batches, each of which included a different assortment of genes (*n* = 98—417 genes, total of 523 genes across all panels). Panels differed as study priorities evolved over the course of RNA processing, leading to some additions or removals. To merge datasets across batches, the following steps were undertaken: 1) batch-specific normalization, 2) probe trimming, 3) concatenation, and 4) imputation, as follows:

### Batch-specific normalization

As described in prior studies [30–33], we used the Removal of Unwanted Variation using control genes (RUVg) function from the RUVSeq package [34] to remove technical effects from each of the 6 individual batches. Separately by batch, corrected expression values were upper quartile normalized and median-centered.

#### Probe trimming

Across batches, a total of 523 genes were profiled (including immune response [*n* = 48] [31], DNA repair [*n*= 54] [32], P53 dysfunction [*n* = 48] [35], PAM50 subtype [*n* = 50] [36], the OncotypeDx/21-gene assay [*n* = 21] [33], and other biologic pathways of interest). All batches included a common core set of 27 genes [5 housekeeping, 22 endogenous] to facilitate batch correction, plus an array of 71—390 additional genes, depending on batch. Fifteen of the 22 common endogenous genes came from the PAM50 algorithm, 10 of which represented proliferation (of the 11 total proliferation genes on the PAM50 panel). To represent a wider variety of biologic pathways, we therefore trimmed our dataset to a larger 219 gene list (Supplemental Table 1) assessed in 4 or more batches of data (> 80% of samples). This set included all genes from the immune, DNA repair, P53, PAM50, and OncotypeDx panels.

### Concatenation

Median-centered expression values for the 219 selected genes were concatenated to yield a single dataset. For samples represented in multiple batches, we retained the specimen with the highest number of genes empirically measured.

### Imputation

Missing values (i.e. for genes not represented in specific batches) were imputed using K-Nearest Neighbors with k = 35, the square root of the number of non-missing observations [37]. This resulted in a 2783 [samples] × 219 [genes] matrix of complete expression values. After imputation, we visualized the 219 genes as well as the common set of 27 genes to shift in principal component (PC) overlays (as evidence of residual batch effects).

### Molecular covariates

We included a research version of PAM50 molecular subtypes defined on 50 genes [36, 38], which classified tumors as Luminal A, Luminal B, HER2-enriched, Basal-like, or Normal-like. RNA-based P53 subtypes were determined based on the previously validated P53-dependent signature, which classifies samples as Mutant-like or Wild-type-like based on a similarity-to-centroid approach [35, 39, 40]. We classified homologous recombination deficiency (HRD High and Low) using 51 DNA repair genes [32]. Immune subtyDistribution of demographic,pes (Adaptive- Enriched, Innate-Enriched, and Immune-Quiet) were previously defined by consensus clustering of the same 48 immune genes used herein [31].

### Statistical analysis

Distributions of non-IBC approximate the distribution of overall population in CBCS (given that only 3% of CBCS were medically-confirmed as IBC). To understand differences in the demographic and clinical characteristics of IBC versus non-IBC cases, we calculated adjusted odds ratio (OR) with logistic regression models. For each demographic or social variable of interest, we fitted two models: adjusted for age and race, which were study selection factors (OR_adj1_), or adjusted for age, race, and HR/HER2 status (OR_adj2_). To identify genes that are significantly differentially expressed by comparing IBC to non-IBC, we performed F-tests from ANOVA on 219 genes. The volcano plot was used to visualize both the significance of these genes and the regulation directions (Down, Unchanged, and Up) respective to log_2_ fold change (log_2_ FC) between IBC and non-IBC. We used Bonferroni Correction to avoid family-wise error, and p-values were log_10_ transformed. A principal component analysis (PCA) plot was included to visualize the first two PCs based on any significantly different genes to observe obvious patterns between IBC and non-IBC. We also performed PCA based on the 48 immune genes, which include markers for multiple immune cells (eosinophils, neutrophils, B-cells, natural killer cells, macrophages, T-cells, CD8 + T-cells, regulatory T-cells, T helper cells, cytotoxic cells, T follicular helper cells, and immune checkpoint), with samples color-coded by immune subtype, to qualitatively assess differences of immune subtype between IBC and non-IBC. All the data used to generate PCA plots were scaled to standardized normal distributions. All of the plots were generated by using ggplot2 package in R [41], and all the statistical analysis for this study was done using SAS software, Version 9.4 of the SAS System for Windows [42].

## Results

### Demographic, clinical, and socioeconomic characteristics

A total of 153 participants in the Carolina Breast Cancer Study were medically-confirmed as IBC. For the 4,586 non-IBC cases (Table 1), 47% self-identified as Black and 51% were under 50 years old. In contrast, for IBC cases, 63% self-identified as Black and 60% younger age. Table 1 shows adjusted ORs, which indicate a significantly increased frequency of IBC among Black and younger women. After adjusting for HR/HER2 status, the race association remained significant [OR 95% CI: 1.94 (1.32, 2.85)], but the age association was no longer significant [OR 95% CI: 1.43 (0.98, 2.07)]. We found that 52% of IBCs had a BMI ≥ 30 kg/m^2^, compared to 43% among non-IBC cases. However, adjusted ORs indicated no significant association with BMI. Similar patterns were observed for participants with a WHR of 0.85 or above (54% had WHR ≥ 0.85 among IBC vs. 46% among non-IBC cases). No significant association was found after adjusting for age, race, or HR/HER2 status. IBC cases were associated with higher grade [III vs. I OR_adj1_ 95% CI: 2.50 (1.47, 4.27); III vs. I OR_adj2_ 95% CI: 2.38 (1.16, 4.89);].

**Table 1:** Distribution of demographic, clinical, and socioeconomic factors among Inflammatory Breast Cancer (IBC) (*n* = 153) vs. non-IBC (*n* = 4,586), Carolina Breast Cancer Study (CBCS) Phases I-III (1993–2013)

	IBC	Non-IBC	OR_adj1_ (95% CI)^a^	OR _adj2_ (95% CI)^b^
	*N* (%)	*N* (%)		
Race				
Non-Black	57 (37%)	2,427 (53%)	REF	REF
Black	96 (63%)	2,159 (47%)	**1.92 (1.38, 2.68)**	**1.94 (1.32, 2.85)**
Age < 50 Years				
No	61 (40%)	2,244 (49%)	REF	REF
Yes	92 (60%)	2,342 (51%)	**1.48 (1.06, 2.05)**	1.43 (0.98, 2.07)
BMI < 30 kg/m^2^				
Yes	72 (47%)	2,578 (56%)	REF	REF
No	79 (52%)	1,961 (43%)	1.24 (0.88, 1.74)	1.32 (0.90, 1.93)
Unknown	2 (1%)	47 (1%)	–	–
WHR < 0.85				
Yes	66 (43%)	2,421 (53%)	REF	REF
No	82 (54%)	2,093 (46%)	1.37 (0.97, 1.92)	1.41 (0.96, 2.07)
Unknown	5 (3%)	72 (2%)	–	–
Tumor Grade				
I	17 (11%)	1,095 (24%)	REF	REF
II	48 (31%)	1,594 (35%)	**1.81 (1.04, 3.18)**	1.90 (0.93, 3.87)
III	88 (58%)	1,897 (41%)	**2.50 (1.47, 4.27)**	**2.38 (1.16, 4.89)**
Below 200% of Poverty Level				
No	69 (45%)	2,812 (61%)	REF	REF
Yes	71 (46%)	1,496 (33%)	**1.61 (1.12, 2.31)**	1.47 (0.98, 2.19)
Unknown	13 (9%)	278 (6%)	–	–
Education Level				
> High School	91 (59%)	2,970 (65%)	REF	REF
<= High School	62 (41%)	1,615 (35%)	1.20 (0.86, 1.69)	1.10 (0.75, 1.61)
Unknown	0 (0%)	1 (0%)	–	–
Urban/Rural				
Urban	32 (21%)	3,887 (85%)	REF	REF
Rural	121 (79%)	699 (15%)	**1.53 (1.03, 2.28)**	1.47 (0.93, 2.32)

^a^ Adjusted for age and race

^b^ Adjusted for age, race, and HR/HER2 status

Several socioeconomic factors revealed significant associations with increased risk of IBC. Income below 200% poverty level, the threshold for government assistance programs, was associated with IBC [OR_adj1_ 95% CI: 1.61 (1.12, 2.31)], but not after adjusting for clinical subtype. Similarly, rural residence was significantly associated with IBC (79% vs. 15% in non-IBC) after adjusting for age and race [OR 95% CI: 1.53 (1.03–2.28)], but not clinical subtype. No significant association was found between education level and IBC prevalence.

### Molecular characteristics

HR/HER2 receptor status differed significantly between IBC and non-IBC cases (Table 2). Adjusted ORs indicated that HER2-positive and triple-negative breast cancers were associated with IBC (compared to HR +/HER2-negative breast cancer). Both HER2-enriched and Luminal B PAM50 subtypes were associated with IBC (compared to Luminal A subtype). However, a slightly lower proportion of IBCs were Basal-like (23% vs. 29% among non-IBC). Mutant-like P53 subtype was strongly associated with IBC [OR 95% CI: 1.79 (1.10, 2.92) vs. Wild-type-like]. In addition, HRD High was associated with IBC [OR 95% CI: 1.90 (1.17, 3.11) vs. HRD Low]. The prevalence of Innate-Enriched immune subtype was slightly higher among IBC (38%) than non-IBC (33%); however, this association was not statistically significant.

**Table 2:** Molecular characteristics of Inflammatory Breast Cancer (IBC) (*n* = 74) vs. non-IBC (*n* = 2,696), Carolina Breast Cancer Study (CBCS) Phases I-III (1993–2013)

	IBC	Non-IBC	OR (95% CI) ^a^
	*N* (%)	*N* (%)	
HR/HER2 Status ^b^			
HR +/HER2-	42 (27%)	2,524 (55%)	REF
HER2 +	41 (27%)	646 (14%)	**3.55 (2.28, 5.52)**
HR-/HER2-	38 (25%)	1,062 (23%)	**1.81 (1.15, 2.85)**
Unknown	32 (21%)	354 (8%)	–
PAM50 Subtypes			
Luminal A	9 (12%)	811 (30%)	REF
Basal-Like	17 (23%)	786 (29%)	1.55 (0.68, 3.54)
Her2-Enriched	23 (31%)	296 (11%)	**6.14 (2.79, 13.5)**
Luminal B	18 (24%)	499 (19%)	**2.90 (1.29, 6.53)**
Normal-Like	7 (9%)	304 (11%)	2.02 (0.75, 5.49)
P53			
Wild-type-like	27 (36%)	1,462 (54%)	REF
Mutant-like	47 (64%)	1,234 (46%)	**1.79 (1.10, 2.92)**
HRD			
HRD Low	27 (36%)	1,507 (56%)	REF
HRD High	47 (64%)	1,189 (44%)	**1.90 (1.17, 3.11)**
Immune Subtypes			
Immune-Quiet	22 (30%)	956 (35%)	REF
Adaptive-Enriched	24 (32%)	840 (31%)	1.07 (0.59, 1.93)
Innate-Enriched	28 (38%)	900 (33%)	1.26 (0.71, 2.22)

^a^ Adjusted for age and race

^b^ The distributions of HR/HER2 Status were calculated for 153 IBC and 4,586 non-IBC

**Fig. 1:**
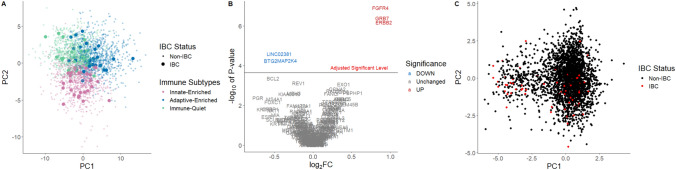
Differential gene expression comparing IBC vs. non-IBC. **A** All three immune subtypes (Immune-Quiet in green, Innate-Enriched in pink, and Adaptive-Enriched in blue) included IBC (larger circles) **B** Only six genes were above the Bonferroni Adjusted significance level in a Volcano Plot of 219 Genes (P-values were -log10 transformed); gray represents non-significant genes, with genes that were down-regulated in IBC in blue, and upregulated in red. **C** PCA plot of the 6 significant genes identified from the volcano plot **B**, colored by IBC (red) vs non-IBC (black) shows no strong differential in first two principal components

To further explore gene expression differences between IBC and non-IBC, we performed several unsupervised and supervised analyses (Fig. 1). First, we visualized the first two PCs across the 48 immune-related genes (Fig. 1A). We observed no strong separation of IBC from non-IBC, and IBCs evenly distributed among all three immune subtypes. Then, we performed differential gene expression analysis. The Volcano Plot (Fig. 1B) showed that among the 219 gene set, only 6 genes showed statistically significant differences (IBC vs. non-IBC after Bonferroni Correction). Three were downregulated (BTG2, LOC400043, and MAP2K4) and three were upregulated (ERBB2, FGFR4, and GRB7) in IBC compared to non-IBC. Notably, the downregulated genes are associated with wild-type-like p53, while upregulated genes were linked to HER2 signaling. The PCA plot of these 6 significant genes (Fig. 1C) showed no obvious separation of IBC from non-IBC.

## Discussion

This study examined demographic and molecular features associated with IBC within a breast cancer study that oversampled Black and younger participants who tend to have higher risk of IBC [2, 17–21]. We confirmed that IBC was more prevalent among Black women with breast cancer. Whereas previous studies have suggested high BMI is correlated with IBC [43–45], we observed no association with high BMI and WHR after adjusting for demographic and clinical factors. We observed associations with PAM50 subtypes, with higher HER2-enriched and Luminal B in IBC vs. non-IBC, and lower Luminal A and Basal in IBC compared to non-IBC. Mutant-like P53 status was strongly associated with IBC as well as high HRD. No association was found with global immune patterns. IBC was also associated with demographic and socioeconomic factors, notably poverty status and rural residence. These findings underscore the importance of health care access for timely diagnosis and treatment of IBC.

Our findings align with previous molecular findings. Van Laere et al. (2013) also found IBC was associated with a higher prevalence of HER2-enriched and lower Luminal A subtypes compared to non-IBC [11]. Other studies also found a higher proportion of Luminal B subtype among IBC compared to other subtypes, although IHC-based subtyping was used to define Luminal B cancers [46–49]. In another recent study, van Geel et al. (2025) found Luminal A subtype was the most prevalent (35%) in IBC [50]. We observed fewer Basal-like (although not significantly) cases among IBC, which is in contrast to Hamida et al. (2008) where they found 33.8% basal cases in IBC vs. 15.9% in non-IBC. However in that study, triple negative status was used to identify putative basal-likes [51]. In addition, the actual frequency differences between IBC and non-IBC across all PAM50 subtypes were very low, suggesting instability (mutant-like P53 subtype and high HRD) in effect estimates may account for these discrepancies.

While few studies have evaluated specific DNA repair pathways, our findings that IBC has more p53 mutations (64% for IBC vs. 46% for non-IBC) concurs with previously published findings (57% to 75% of IBC cases had TP53 mutation) [52–55]. We observed a moderate association between IBC and HRD, and Rypens et al. (2024) proposed genomic instability as a hallmark of IBC [10]. In contrast, Bertucci et al. (2024) did not find a difference in continuous HRD score between IBC and non-IBC [56]. These differences may be attributed in part to our gene expression-based approach which focuses on pathway dysregulation, whereas their HRD score was based solely on DNA differences [56]. In addition, the use of continuous vs. categorical data may make direct comparisons challenging.

Results in this study differ from some previous literature focusing on immune characteristics of IBC. Some of these previous studies found enriched expression of B-cells, M2 macrophages, regulatory T-cells, CD8 + and CD4 + T-cells, and immune-checkpoints such as PD-L1 among IBC compared to non-IBC [11, 57–61]. In contrast, our study found that neither global immune classes nor the expression of individual immune genes were associated with IBC. This lack of differential immune gene expression may suggest that, contrary to earlier reports, immune modulation may not play a central role in shaping IBC, or alternatively, that more refined immune classifiers are needed to detect biologically relevant differences. We acknowledge that limited statistical power may partly explain the null associations observed for immune subtypes.

Our results suggest that understanding IBC's aggressive nature and concomitant disparities requires looking beyond molecular profiles to demographic and social correlations. Our finding of higher IBC prevalence in Black women aligns with prior research [2, 16–20]. We also found IBC was more prevalent among younger breast cancer participants, which aligns with prior documentation of more aggressive breast cancer subtypes in younger women [62–64]. While previous literatures showed consistent crude association between BMI and IBC [21, 43–45], few studies adjusted for age, race, or breast cancer subtypes. Our findings imply that the association of obesity and IBC may vary by demographic or other molecular characteristics.

Previously, associations of social factors and IBC showed inconsistent results. Some studies found that comparing IBC to non-IBC showed no difference with regards to education and family income level [43, 65–68]. However, other studies found that IBC is associated with lower education levels, living in an area with higher poverty, and residence in urban areas [44, 69, 70]. Notably, those studies usually had a much higher proportion of white individuals compared to other races (65% to 82% white). Lower prevalence of poorer socioeconomic status in those studies may have resulted in underpowered analysis of some social patterns. Unlike early studies, we found a strikingly higher proportion of IBC participants living in rural areas (79%) compared to non-IBC (15%), and significant association held even after adjusting for race and age.

Several different genes sets have been reported previously that distinguish IBC from non-IBC [11–14]. In CBCS, our analysis of 219 genes identified only six that were differentially expressed between IBC and non-IBC. The three genes that we identified as upregulated in IBC were associated with HER2 signaling, which fits with our observation here and that of others [71] that IBC has a higher rate of HER2 + subtype compared to non-IBC. The three genes that we identified as downregulated in IBC were associated with p53, and included BTG2, a tumor suppressor directly regulated by p53 [72, 73]. The low number of DEGs identified in our study may be due to our gene panel focusing on PAM50, P53, DNA repair, and immune pathways and thus differing from those used in other studies. The small number of IBC (*n* = 74 vs. *n* = 2,696 non- IBC) with molecular data limited our power to detect biologically meaningful differences in gene expression. Chakraborty et al. (2021) previously attempted to validate statistically significant differences between IBC and non-IBC across different validation datasets and found few or no consistencies [15]. Similarly, Funakoshi et al. reported two sets of DEGs between triple-negative-IBC and triple-negative-non-IBC, but both showed high false discovery rates [false discovery rate > 0.2] [16].

Future research should prioritize investigating individual and community-level social determinants and their associations with healthcare access and early diagnosis of IBC. While our study observed certain molecular characteristics associated with IBC, including a higher frequency of HER2-enriched and Luminal B subtypes, mutant-like P53, and high HRD, the overall limited number of IBC cases and significant DEGs, and the lack of a distinct immune profile, failed to replicate results from early studies. However, IBC is a challenging diagnosis with variations in clinical presentation [3, 4]. Common diagnostic criteria are being defined [4] and validated clinically [74]. Therefore, the inclusion of larger numbers of IBC samples in future molecular studies that have been clearly diagnosed as definite IBC may help in identifying unique IBC molecular/genetic signatures. The associations we observed for IBC with rural residence, poverty, younger age, and Black women underscore the importance of early diagnosis and healthcare access. Targeting early detection could help reduce disparities in IBC and improve IBC prognosis.

## Supplementary Information

Below is the link to the electronic supplementary material.

Supplementary file1 (DOC 34 KB)

## Data Availability

CBCS data are available upon request (https://unclineberger.org/cbcs).
