# Supplementary material for: Molecular and socioeconomic characteristics of inflammatory breast cancer in the Carolina Breast Cancer Study
Source: Breast Cancer Res Treat. 2026 Jan 22;215(2):61. doi: 10.1007/s10549-025-07884-3 (PMC12827424; doi:10.1007/s10549-025-07884-3)
Supplement: Supplementary file 1 — Supplementary file1 (DOC 34 KB) [file 10549_2025_7884_MOESM1_ESM.doc]

| **Supplemental Table 1. List of 219 genes** | |
| --- | --- |
| **Gene Signatures** | **Gene Names** |
| **DNA Repair** | APEX1, APOBEC1, APOBEC3A, APOBEC3B, APOBEC3C, APOBEC3D, APOBEC3F, APOBEC3G, APOBEC3H, BRCA1, BRCA2, ERCC5, FANCA, FANCD2, FANCI, FANCL, FEN1, HORMAD1, LIG1, LIG3, MAGEA1, MAGEA10, MAGEA4, MAGEA6, MLH1, MLH3, MRE11, MSH2, MSH3, MSH6, NBN, NEIL1, NEIL2, PARP1, PMS2, POLB, POLH, POLI, POLK, POLQ, PRKDC, RAD18, RAD50, RAD51, REV1, RNF168, RNF8, TP53BP1, UBE2A, UBE2B, XPA, XPC, XRCC1, XRCC5 |
| **Immune** | ALK, BLK, CCL7, CCR3, CD19, CD2, CD274, CD28, CD3E, CD3G, CD4, CD6, CD68, CD84, CD8A, CD96, CXCL13, CXCL5, CXCR1, CXCR2, CXCR5, CYBB, FAM30A, FCRL2, FN1, FOXP3, FPR2, GZMM, HAVCR2, HLA-DOB, ICOS, IL2RB, IL5RA, LAG3, LCK, LILRB2, MAF, MS4A1, MSR1, NCR1, NFKB1, PDCD1, PPBP, PRF1, PTGDR2, SH2D1A, SIRPG, TNFRSF17, TNIK, TRAF1, TRIP13, ZAP70 |
| **OncotypeDX** | AURKA, BAG1, BCL2, BIRC5, CCNB1, CD68, CDC20, CTSV, ERBB2, ESR1, GRB7, GSTM1, MKI67, MMP11, MYBL2, NDC80, NUF2, PGR, RRM2, SCUBE2, TFRC |
| **P53** | APH1B, ATAD2, ATOSA, AURKA, BTG2, CCNA2, CCND1, CDC25B, CDC25C, CDCA7L, CDK1, CDKN1A, CDKN3, CENPF, CEP55, CKS1B, DDB2, FNBP1, FOXM1, GASK1B, GATA3, GGH, KIAA0040, KIF23, KIFC1, LINC02381, MAD2L1, MAP2K4, MCM3, MIS18A, MKI67, MYBL2, NCAPH2, NEO1, NPEPPS, NUDT1, POLD1, PREP, PTTG1, RFC4, RNF103, SLC39A6, TAP1, TCEAL1, TOP2A, TRIP13, TUBA4A, UBE2C |
| **PAM50** | ACTR3B, ANLN, BAG1, BCL2, BIRC5, BLVRA, CCNB1, CCNE1, CDC20, CDC6, CDH3, CENPF, CEP55, CXXC5, EGFR, ERBB2, ESR1, EXO1, FGFR4, FOXA1, FOXC1, GPR160, GRB7, KIF2C, KRT14, KRT17, KRT5, MAPT, MDM2, MELK, MIA, MKI67, MLPH, MMP11, MYBL2, MYC, NAT1, NDC80, NUF2, ORC6, PGR, PHGDH, PTTG1, RRM2, SFRP1, SLC39A6, TMEM45B, TYMS, UBE2C, UBE2T |
| **Housekeeping/Others** | ACOX2, CRYBB2, FAM177A1, GSTT2, MUC1, PSPH, PSPHP1, SQLE, ACTB, CLTC, GAPDH, GUSB, HPRT1, MRPL19, PGK1, PSMC4, RPLP0, SF3A1, TUBB |
